# The Role of miRNAs in Dexmedetomidine’s Neuroprotective Effects against Brain Disorders

**DOI:** 10.3390/ijms23105452

**Published:** 2022-05-13

**Authors:** Codrin-Constantin Burlacu, Maria-Adriana Neag, Andrei-Otto Mitre, Alexandru-Constantin Sirbu, Andrei-Vlad Badulescu, Anca-Dana Buzoianu

**Affiliations:** 1 Faculty of Medicine, Iuliu Hatieganu University of Medicine and Pharmacy, 400349 Cluj-Napoca, Romania; codrinburlacu@gmail.com (C.-C.B.); andrei.mitre97@gmail.com (A.-O.M.); andibadulescu@gmail.com (A.-V.B.); 2 Department of Pharmacology, Toxicology and Clinical Pharmacology, Iuliu Hatieganu University of Medicine and Pharmacy, 400337 Cluj-Napoca, Romania; alexdak.sirbu@gmail.com (A.-C.S.); abuzoianu@umfcluj.ro (A.-D.B.)

**Keywords:** dexmedetomidine, microRNAs, brain injury, neuroprotection, drug-response variability

## Abstract

There are limited neuroprotective strategies for various central nervous system conditions in which fast and sustained management is essential. Neuroprotection-based therapeutics have become an intensively researched topic in the neuroscience field, with multiple novel promising agents, from natural products to mesenchymal stem cells, homing peptides, and nanoparticles-mediated agents, all aiming to significantly provide neuroprotection in experimental and clinical studies. Dexmedetomidine (DEX), an α2 agonist commonly used as an anesthetic adjuvant for sedation and as an opioid-sparing medication, stands out in this context due to its well-established neuroprotective effects. Emerging evidence from preclinical and clinical studies suggested that DEX could be used to protect against cerebral ischemia, traumatic brain injury (TBI), spinal cord injury, neurodegenerative diseases, and postoperative cognitive disorders. MicroRNAs (miRNAs) regulate gene expression at a post-transcriptional level, inhibiting the translation of mRNA into functional proteins. In vivo and in vitro studies deciphered brain-related miRNAs and dysregulated miRNA profiles after several brain disorders, including TBI, ischemic stroke, Alzheimer’s disease, and multiple sclerosis, providing emerging new perspectives in neuroprotective therapy by modulating these miRNAs. Experimental studies revealed that some of the neuroprotective effects of DEX are mediated by various miRNAs, counteracting multiple mechanisms in several disease models, such as lipopolysaccharides induced neuroinflammation, β-amyloid induced dysfunction, brain ischemic-reperfusion injury, and anesthesia-induced neurotoxicity models. This review aims to outline the neuroprotective mechanisms of DEX in brain disorders by modulating miRNAs. We address the neuroprotective effects of DEX by targeting miRNAs in modulating ischemic brain injury, ameliorating the neurotoxicity of anesthetics, reducing postoperative cognitive dysfunction, and improving the effects of neurodegenerative diseases.

## 1. Introduction

Brain injury refers to a multitude of conditions, which are often life-threatening, lead to chronic disability, and subsequently high socioeconomic costs, while therapeutic strategies often have limited effectiveness. Of this wider category, the most debilitating are traumatic and ischemic brain injuries, with neuroprotective agents most commonly used in this setting [1]. Neurodegenerative disorders including Alzheimer’s disease (AD), Parkinson’s disease (PD), multiple sclerosis (MS), amyotrophic lateral sclerosis (ALS), and Huntington’s disease (HD), which are responsible for long-term disability and cognitive impairment, have become a growing cause of morbidity and mortality in both elderly and young patients [2,3]. Neuroinflammatory processes contribute to the initiation and progression of these neurodegenerative diseases, as well as to neurodegeneration and postoperative cognitive impairments related to surgical stress and anesthetics toxicity [4,5,6].

Neuroscience confronts with limited neuroprotective strategy for multifold CNS conditions, for which a fast and sustained management, initially established is essential. Neuroprotection-based therapeutics have become an intensively researched topic in the neuroscience field, with multiple novel promising agents, from natural products to mesenchymal stem cells, homing peptides, and nanoparticles-mediated agents, all aiming to significantly provide neuroprotection in experimental and clinical studies [7,8,9,10]. Despite a growing understanding of this process, the vast majority of agents that showed promising effects in preclinical studies have failed to demonstrate a clear clinical benefit [11].

In this context, dexmedetomidine (DEX), typically used as a sedative, anesthetic adjuvant, and opioid-sparing medication, stands out due to its established neuroprotective effects, which seem to translate well from animal models to a clinical setting [12,13,14]. While its applications in anesthesiology and pain management are consistent with its pharmacological activity as a selective α2-adrenoceptor agonist, the precise mechanisms of DEX-induced neuroprotection are mostly unclear. In addition to the neuroprotective effects of its sympatholytic, α2-agonist, activity, several neuroprotective mechanisms confirmed in animal brain disease models involve anti-inflammatory effects through the inhibition of TLR4/NF-κB, JAK2-STAT3, activation of PI3K/Akt and ERK1/2 pathways, and inhibition of apoptosis and autophagy through hypoxia-inducible factor 1-α (HIF-1α) upregulation [12,15,16,17,18,19].

MicroRNAs (miRNAs), small (18–22 nucleotides), non-coding ribonucleic acid (RNA) molecules, regulate gene expression at the post-transcriptional level, by interacting with the 3′ UTR of mRNAs, thus silencing the translation of mRNA into functional proteins [20,21]. miRNAs regulate a variety of biological processes in a cell-specific manner, including stem cell proliferation and cell differentiation, apoptosis, as well as malignancy, cardiovascular disease, neurodegeneration, and autoimmunity [22,23].

In vivo and in vitro studies deciphered brain-related miRNAs and dysregulated miRNA profiles after several brain disorders, including ischemic stroke, AD, MS, and brain tumors, emerging new perspectives in neuroprotective therapies by miRNAs modulation [24,25,26,27].

Experimental studies revealed that some of the neuroprotective effects of DEX are mediated by various miRNAs, counteracting multiple mechanisms in several disease models, such as neuroinflammation induced by lipopolysaccharides (LPS), β-amyloid, brain ischemic-reperfusion injury and anesthesia-induced neurotoxicity models [28,29,30,31].

On the other hand, miRNAs can greatly influence drug response, either by modulating pharmacokinetics and pharmacodynamic, or by influencing the pathological process itself [32]. In the case of DEX, multiple clinical studies revealed changes in miRNA expression upon DEX treatment, associated with different pharmacology features of DEX [33].

An in-depth understanding at a molecular level of the mechanistic data of DEX in the context of neuroprotection and the variability of DEX response will further provide new perspectives on neurotherapeutic strategy. This review aims to outline the neuroprotective mechanisms of DEX in brain disorders by modulating miRNAs, alongside the miRNAs involved in the pharmacological profile of DEX. We address the neuroprotective effects of DEX by targeting miRNAs in modulating ischemic brain injury, ameliorating the neurotoxicity of anesthetics, reducing postoperative cognitive dysfunction, and improving the effects of neurodegenerative diseases.

## 2. Pharmacological Features of Dexmedetomidine

DEX is the S-enantiomer of medetomidine, a sedative agent used in veterinary medicine [34]. It was first approved as an FDA drug in 1999 and was used as a sedative in patients in the intensive care unit (ICU). Subsequently, in 2011, DEX was approved by the European Union. Nowadays, this substance is a well-known drug used for sedation and anesthesia [35].

It acts as an agonist with a high affinity for α2 adrenergic receptors (AR) [36]. Like clonidine, DEX is an imidazole derivate, but has a higher selectivity to α2 receptors than this [34,37]. There are three types of α_2_AR (α_2A_, α_2B_, α_2C_) that are important targets in the treatment of pain or diseases such as high blood pressure, hyperactivity disorder, etc. [38].

More than 90% of the DEX administered is protein bound. DEX is metabolized in the liver by hydroxylation and glucuronidation by CYP2A6, UGT2B10, and UGT1A4. The resulting metabolites are considered inactive, being eliminated mainly renally and a small percentage in the feces [39]. Notably, the pharmacokinetics of dexmedetomidine is not influenced by patients’ renal function, allowing DEX anesthesia in patients with renal failure [40,41].

The sedative effect depends on the plasma concentration. At low plasma concentrations (0.2–0.3 ng/mL) DEX causes mild/moderate sedation, while at high levels (1.9 ng/mL) it is responsible for deep sedation [39].

Drug interactions may occur when DEX is used concomitantly with other medicines. Butorphanol, as an opioid analgesic, exerts antinociception and sedation via acting on MORs, KORs, and DORs, but when used concomitantly with DEX, could suggest a synergistic antinociception and sedation activity in the treatment of acute nociceptive pain [42]. Parecoxib, a cycloxygenase-2-inhibitor, commonly used as a perioperative clinical anesthetic agent in combination with DEX, is rapidly converted in Valdecoxib, its active metabolite [43,44,45]. As DEX inhibits cytochrome P450 and parecoxib’s metabolism depends on CYP3A4 and CYP2C9, cotreatment with DEX and parecoxib will further lead to an inhibition on valdecoxib’s metabolism, its active metabolite, and subsequent increased exposure time to valdecoxib [46].

Alongside propofol and midazolam, DEX is widely used for sedation of critically ill patients who are undergoing mechanical ventilation. Compared to these, DEX does not act through gama aminobutyric acid (GABA) pathway like propofol and midazolam [36]. DEX has sedative and analgesic effects with a different mechanism of action compare with opiates, a well-known class of drugs with analgesic effects [47].

One of the major advantages of DEX compared with GABA receptor agonists is its safety profile against respiration depression [48]. Another advantage of DEX is that if it is given concomitantly with opiates it reduces the required dose of opiates in ICU patients [49].

Among α2-agonists, DEX has shown to have a greater efficacy than clonidine as premedication for bupivacaine spinal block and rarer hemodynamic adverse effects (hypotension and rebound hypertension) than clonidine for the sedation of ICU patients requiring endotracheal intubation [50,51].

Multiple clinical studies evidenced the potential of perineural DEX, used in spinal anesthesia and brachial plexus block, to prolong the motor and sensory block, as well as time to first analgesic request, suggesting DEX as an alternative to opioids [52].

The main side effects of DEX are hypotension and bradycardia. They often have no clinical impact and do not require treatment. However, if treatment is needed, dose reduction of DEX, and administration of fluids or vasopressors are recommended [53]. In a meta-analysis including 516 patients from nine clinical trials, Abdallah et al., have been shown reversible bradycardia, with no hypotension and respiratory depression, after intrathecally or peripherally infusion of DEX [52].

The main effects of DEX are mentioned in Table 1.

The beneficial effect of DEX is dose-dependent. For example, after general anesthesia, there are many unwanted symptoms such as high blood pressure, tachycardia, agitation, shivering, or coughing. Thus, different doses of DEX (1 µg/kg, 0.5 µg/kg, and 0.25 µg/kg), administered in bolus at the end of the surgery, were tested to observe the reduction of the above-mentioned symptoms. The results showed that a higher dose (1 µg/kg) significantly decreases all symptoms, the middle dose (0.5 µg/kg) was effective, except for cough, and the lower dose (0.25 µg/kg) caused hypotension, but did not delay extubation [65].

Multiple disease models revealed a different miRNA expression profiling upon DEX treatment, suggesting the role of miRNAs in the organ-protective effects of DEX (Table 2). Therefore, some of the cytoprotective effects of DEX are mediated by various miRNAs, counteracting multiple mechanisms in several disease models, neuroinflammation induced by LPS and β-amyloid, lung injury in chronic obstructive pulmonary disease, and ischemia/reperfusion injury in the myocardium and liver [28,29,66,67,68].

We performed KEGG pathway analysis using miRNet and identified the following pathways as being influenced by the upregulated miRNAs upon DEX treatment: Neurotrophin signaling pathway (adjusted *p*-value = 3.27 × 10^−13^), T cell receptor signaling pathway (adjusted *p*-value = 1.73 × 10^−9^), MAPK signaling pathway (adjusted *p*-value = 7.37 × 10^−9^), p53 signaling pathway (adjusted *p*-value = 1.25 × 10^−7^), Chemokine signaling pathway (adjusted *p*-value = 6.3 × 10^−7^), apoptosis (adjusted *p*-value = 5.4 × 10^−6^), Jak-STAT signaling pathway (adjusted *p*-value = 6.19 × 10^−6^), Toll-like receptor signaling pathway (1.72 × 10^−5^), axon guidance (adjusted *p*-value = 2.35 × 10^−5^), VEGF signaling pathway (adjusted *p*-value = 7.38 × 10^−5^), mTOR (adjusted *p*-value = 1.64 × 10^−4^), Wnt signaling pathway (adjusted *p*-value = 6.93 × 10^−4^), TGF-beta signaling pathway (adjusted *p*-value = 2.6 × 10^−3^); Leukocyte transendothelial migration (adjusted *p*-value = 7.5 × 10^−3^), NOD-like receptor signaling pathway (adjusted *p*-value = 9.56 × 10^−3^), and Natural killer cell mediated cytotoxicity (adjusted *p*-value = 1.2 × 10^−2^).

According to KEGG pathway analysis, the common role in the DEX-upregulated miRNAs is represented by anti-inflammatory, anti-apoptotic, and oxidative stress-suppressing signaling pathways, which provide neuroprotection in brain injury models. The neuroprotection effects of DEX by influencing miRNAs are mediated by all these pathways retrieved by KEGG pathway analysis and will be further described in this review.

Some of the most essential miRNAs listed in Table 2, which are involved in DEX-induced neuroprotection are represented by miR-7-5p, miR-214-5p, miR-29a-3p, and miR-381. They directly mediate neuroprotective effects of DEX and also strengthen the beneficial effects of DEX in different brain disease models. The other microRNAs pose an indirect role in DEX-induced neuroprotection, indirectly mediating several neuroprotective pathways.

## 3. Evidence of Dexmedetomidine as a Neuroprotective Agent

Even if DEX has been successfully used as an agent for sedation and anesthesia, more and more studies are pointing towards its potential use as a neuroprotective agent. Its role in neuroprotection has been underlined in several experimental and clinical studies, and even though there are several explanations for the mechanisms that are not perfectly understood. The neuroprotective effects of DEX have been ascertained in multiple animal models of brain injury, such as ischemic brain injury, traumatic brain injury (TBI), neurodegenerative diseases, and postoperative cognitive disorders [13].

The main neuroprotective mechanism, resulted from the intrinsic sympatholytic activity of DEX as α2-agonist, consisting of negatively regulation of catecholamine releasing, by activation of α2AR in locus coeruleus, thus hampering cerebral vascular spasms and brain damage [84].

Studies have so far observed that DEX reduces pro-inflammatory cytokines. Previous experimental studies have proven that it reduces interleukin 6 (IL-6) release through Interleukin 1-β (IL1-β) suppression [85,86]. DEX also attenuates neuroinflammation through the cholinergic anti-inflammatory pathway, enhancing the discharge of the cervical vagus, thus decreasing the systemic cytokine levels [87]. Another neuroprotective effect is proceeded by reducing pro-inflammatory effects of TNF-α and nuclear factor kappa-light-chain enhancer of activated B cells (NF-κB) [88]. By binding to α2 receptors of neurons, DEX reduced the release of catecholamines and modulated receptors’ sensitivity and release of glutamate. Glutamate release is also reduced by the suppression of voltage-dependent calcium channels but also by activated protein kinase activity [89]. DEX also enhances the expression of the excitatory amino acid transporters 1 and 3, glutamate transporters that remove glutamate from the synaptic cleft and extra synaptic sites by reuptake in neurons and glial cells [90,91]. Otherwise, DEX could inhibit extracellular glutamate by hampering the response of glutamate channels to depolarization and could also inhibit the neurocyte’s absorption ability of glutamate [84].

In response to apoptosis, DEX modulates pro and anti-apoptotic proteins, including Bcl-2 and Bcl-xl [92]. Neuronal hypoxic damage can cause adaptive reactions by increasing the HIF-1α and the vascular endothelial growth factor (VEGF) to protect the neuronal cell from oxygen and glucose depravation (OGD). By binding to the I2 imidazoline receptor, DEX upregulates the expression of both, VEGF and HIF-1α, promoting neuroprotective effects [93]. Other neuroprotective mechanisms of DEX consist of anti-inflammatory effects by inhibiting TLR4/NF-κB, JAK2-STAT3 pathways, and anti-apoptotic effects by activating PI3K/Akt, ERK1/2 pathways, and anti-apoptotic and -autophagic effects through HIF-1α upregulation [12,15,16,17,18].

Besides the experimental evidence that supports the neuroprotection of DEX, there are also several clinical studies that confirm its benefits in the clinical settings. In clinical randomized trials, DEX provided neuroprotection in septic patients, improving significantly the mortality rates up to 70% compared to lorazepam sedated patients, and has also reduced the incidence and duration of delirium in elderly ICU patients, candidates for non-cardiac surgeries, compared to placebo [94,95,96]. These might be explained by the different immune-modulatory profiles of anesthetic agents. Compared to benzodiazepines and midazolam, which impaired neutrophils and macrophages activity, DEX exerts stimulatory effects on macrophage phagocytosis, with no influence on neutrophil activity, thus exerting protective effects [97,98]. Moreover, in the prophylaxis of postsurgical delirium in elderly patients after coronary artery bypass grafting, the DEX treatment group had lower mortality, a shorter ICU stay, and a lower opioid consumption [99].

In 160 patients undergoing elective cranial surgery, DEX decreased postoperative neurological complications and postoperative delirium. or saline combined with goal-directed hemodynamic therapy to evaluate the neuroprotective effect [100]. DEX sustain recovery of cognition functions in 49 patients after carotid endarterectomy, lowering cerebral neuroinflammatory markers, IL-6, TNF-α, lactate content, and increasing brain-derived neurotrophic factor (BDNF) levels after 24 h of the procedure [101]. Blood samples of 60 glioma patients who underwent craniotomy revealed a decreased level of IL-6, TNF-α, neuron-specific enolase, S100 β, malondialdehyde, and increased level of superoxide dismutase after DEX pretreatment, suggesting the anti-inflammatory and anti-oxidative role of DEX against brain damage [102].

## 4. Short Overview of miRNAs as Therapeutic and Diagnostic Tools in Brain Disorders

In the recent years, increasing evidence highlights the regulatory roles of miRNAs behind protein expression in developmental processes such as: stem cell division, cell differentiation, proliferation, and apoptosis, defining new mechanisms involved in different pathological conditions, including cancer, cardiovascular, neurodegenerative diseases, immune system disorders [22,23].

The first well-documented disease, where miRNAs have been shown to play a regulatory role was chronic lymphocytic leukemia, with the extension of the area of experimental research in multiple pathological settings: cancer, cardiovascular, autoimmune disorders, and neurological disorders [22,103]. Extracellular miRNAs have been found in a remarkably stable state in serum or plasma, resistant to RNase and unfortunate physical conditions due to different carriers such as microvesicles and exosomes that mediate miRNA transport [104,105]. Thus, they might serve as potential clinical biomarkers, for multiple conditions including cancer, diabetes, cardiovascular diseases, and neurodegenerative diseases [106].

In neuroscience, miRNAs gained attention in neurological disorders as emerging new promising biomarkers to estimate the progression and extension of multiple disorders, as well as new therapeutic strategies aimed to protect the brain and sustain recovery from brain damage [107,108]. Certain blood miRNAs emerged as biomarkers for ischemic stroke, intracerebral hemorrhage, and seizures, such as: miR-210, -125a-5p, -miR-155, and miR-362-3p, -125b-5p, miR-143-3p, -miR-298 [109,110,111].

Ponnusamy et al., deciphered the role of miRNAs involved in normal brain development, including the neurodevelopment processes of myelination and synaptogenesis, vasculo-genesis, angiogenesis then continuing with normal functioning processes of the brain of synaptic plasticity and memory [112].

Following hypoxic/ischemic injury, miRNAs modulate a chain of inflammatory responses, leading to microglial activation, cytokine production, apoptosis, energy balance, and cell apoptosis; all these events are influenced by miRNAs [24].

MiRNAs dysregulation profile of miRNAs has been ascertained in ischemic brain injury, TBI, neurodegenerative diseases, i.e., AD, HD, PD, prion diseases, brain tumors, cerebral infections, and hypoxic-ischemic encephalopathy [113,114]. As key mediators in multifold pathological conditions, miRNAs have been widely exploited as theragnostic tools in cerebral ischemia, further providing a promising neuroprotective strategy in ischemic stroke. Using microarray and stem-loop Real-Time PCR analysis to measure miRNA expression, of the 157 miRNAs found to be regulated in 19 patients with ischemic stroke, 138 miRNAs exhibited upregulated levels and 19 miRNAs exhibited downregulated levels [115].

MiRNAs expression profiling has been shown to change significantly in a time-dependent manner. For example, miR-290 and miR-494 exhibited differential changes in their expression at 24 and 48 h after the stroke event, while miR-150, -195, and -320 reversed their expression profiling compared to the previous ones. The number of dysregulated miRNAs decreased significantly from 106 after 24 h of stroke induction to 82 after 48 h of stroke event, suggesting the dynamic of miRNAs expressing profiling in promoting neuroprotective mechanisms of the ischemic brain [116]. The level of miR-223-3p, miR-155-5p, miR-3473, and miR-448-5p in the blood serum, liver, and spleen, have been changed in time-dependent manner in ischemic stroke rat model, with differential expressed miRNAs in the brain between different anatomical areas [117].

MiRNAs biosynthesis complexes are influenced by pathological conditions. For instance, Ago and Dicer enzymes have been shown to be upregulated upon ischemic insult after 24 h and Dicer, XPO5, and TRBP increased in the spleen after 72 h [117]. Moreover, miRNA–RISC complex configurations are modulated by ischemic/hypoxic conditions [118].

Understanding the involvement of miRNAs in the complex array of a neuropathophysiological chain of reactions reveals new potential therapeutic targets of miRNAs in brain injuries. Using bioinformatics analysis, Chenjing et al., mapped the dysregulated profile of miRNAs in AD, consisting of 27 dysregulated miRNAs, involved in pathogenesis processes of AD, i.e., amyloidogenesis, inflammation, neurite differentiation, degradation of neurons, and neuronal survival and proliferation [119]. In preclinical models of intracerebral hemorrhage, several miRNAs have been related to beneficial neuroprotective effects, such as miR-223,-7, -let-7a, -23b, -126-3p, -132, -140-5p, -146a, -152, -181c, -183-5p and -194-5p, whereas others have been associated with deleterious effects, including miR-222, -494, -23a-3p both acting on neuroinflammatory signaling pathways [120].

MiRNAs-based agents—agomirs and antagomirs—chemically modified oligonucleotides modulate target miRNAs, by replacing or silencing miRNAs, depending on the ‘regulation’ state of these miRNAs. In this way, we can replace a low concentration of a beneficial miRNA or inhibit a highly expressed miRNA related to deleterious effects, using these agents [107,121]. Thus, miR-153 and miR-205 have been identified as potential therapeutic targets in PD, as miR-153 and miR-205 mimic targeting a-synuclein and LRRK2 protein in PD [122,123].

Otherwise, blocking the expression of some miRNAs involved in detrimental effects of brain injury processes could be used as a therapeutic strategy [124]. For instance, intracerebroventricular administration of anti-miR-103-1 in the ischemic stroke rat model upregulates the expression of Na^+^/Ca^2+^ exchanger, which was related to the maintaining of ionic homeostasis in the ischemic brain [125]. MiR-181a antagomir provides a new promising therapeutic strategy in stroke patients for long-lasting recovery of motor function and coordination.

Notably, the miRNAs expression profiling changes during pathological events depending on the intensity of the injurious stimulus. For instance, miR-181a expression level was upregulated in ischemic stroke tissue, increased levels being associated with increased injury and oxidative stress [126]. In the penumbra zone of ischemic stroke, miR-181a has been shown to be downregulated, thus protecting neurons and glial cells [127]. By the same mechanism, ischemic/hypoxia preconditioning downregulated miR-181a expression in oxygen-glucose deprivation/reperfusion (OGD/R) neuron model and MCAO rat model, protecting cerebral tissue against ischemia/reperfusion (I/R) injury, by inhibiting apoptosis and pyroptosis of neurons and enhancing the protective function of neuronal mitochondria [127].

## 5. Regulation Production of miRNAs after Dexmedetomidine Treatment

### 5.1. miRNA Biogenesis

The multistep process of miRNA biogenesis begins with DNA transcription of the miRNA-coding genes from introns of protein-coding genes and intergenic miRNAs regions. This will further lead to pri-miRNA transcript, and the formation of pre-miRNA [21,128]. Then, the pre-miRNA is exported into the cytoplasm and processed by RNase III Dicer, producing the mature miRNA duplex [129]. Formation of the RNA-induced silencing complex (RISC), the silencing inductors of mRNA, requires loading the miRNA duplex into the Argonaut (AGO) family of proteins [21,130]. By base pairing the 5′ end with 3′ untranslated regions (3′-UTRs) of mRNAs, 7-methylguanosine from mRNA complex interacts with Ago proteins from the RISC complexes of miRNAs, inducing miRNA-mediated gene silencing [131,132]. The formation process of miRNAs is mediated by specific enzymes: RNA polymerase II, micro-processor complex (Ribonuclease III enzyme, Drosha, and DGCR8), Exportin 5 transporter, RNase III Dicer, and Ago2 [21,133]. Depending on what region has bound, miRNAs could induce both silencing gene expression, by binding to 5′ UTR or coding regions or inducing transcription by the interaction with the promoter region [134,135].

### 5.2. DEX-Dependent Transcription Controlling Systems of the miRNAs

MiRNAs biosynthesis complexes are influenced by several pathological conditions. Under hypoxic conditions, manifold mechanisms that regulate miRNA biogenesis machinery are triggered at different levels: transcription, maturation, and function levels [136].

Different molecular mechanisms might be involved in DEX-induced signals on miRNAs biogenesis. Hypoxia, which contributes to the pathogenesis of ischemic-hypoxic brain injuries, neuroinflammatory diseases, and neurodegenerative diseases, has a direct impact on enzymatic complexes of miRNA biogenesis [136,137]. By inducing the expression of HIF, p53, and NF-kB pathway, hypoxia could stimulate miRNA transcription, thus influencing the expression level of miRNAs [136]. Multiple anti-inflammatory effects of DEX proceed via regulating the NF-kB signaling pathways and EGR1/p53 pathway [70,74]. By modulating NF-kB and p53-associated pathways, DEX might interfere with the synthesis process of the altered brain-related miRNA in different brain disorders associated with hypoxic conditions. Drosha, DRG8 complexes are suppressed under hypoxia, thus inhibiting the cleavage of pri-miRNA into pre-miRNA [117,136]. Moreover, miRNA–RISC complexes are stimulated by hypoxia, leading to mRNA degradation and translation repression in brain injuries [136]. Therefore, DEX might counteract the inhibitory or stimulatory effects of hypoxia on miRNA biogenesis complexes, which might lead to favorable effects in brain disorders. α and β AR activate transcription of IL-6, and TNF-α in vivo astrocyte cultured models of rat spinal cord, suggesting the potential of adrenoceptors to induce transcription activation of different factors [138,139]. Dexmedetomidine poses transcriptional activation roles in astrocyte cultures, increasing TNF-α mRNA, which leads to JNK and ERK phosphorylation [139].

DEX, α2 AR agonist, by binding to G-protein-coupled receptors (GPCRs) potentiates guanine-nucleotide regulatory binding proteins (G inhibitory protein), which leads to decreased cAMP level. [13] By affecting GPCRs, DEX might influence the miRNA expression, by controlling the transcription systems of miRNA.

Multiple studies revealed that miRNAs are affected after stimulating G-protein-coupled receptors (GPCRs), several miRNAs acting as downstream targets of GPCRs [140]. In breast cancer and hepatocarcinoma, miR-144 is regulated by GPER via modulating PI3K/ERK1/2/Elk1 pathway [141]. miR-148a acts as a bidirectional modulating molecule, as E2-GPER downregulates miR-148a and miR-148 downregulates HOTAIR, non-coding RNA [142].

The controlling systems that affect different steps in miRNA synthesis, maturation, and function after DEX treatment are not fully understood. The research studies mentioned in this review are focused on the level of expression of miRNAs after DEX administration, specific mRNA targets of miRNA, and associated biological effects.

More research studies to depict the biological systems that affect the up- and down-regulation of miRNAs in response to DEX treatment will be needed.

## 6. Translation Relevance of miRNA in Dexmedetomidine-Mediated Neuroprotection

### 6.1. Neuroprotective Profile of Dexmedetomidine in Brain Disorders

Over the last years, the exploring of multiple neuroprotection agents for targeting brain damage tissue in experimental animal studies, turn out unsuccessfully from bench to bedside in brain disorders research [143]. Emerging evidence from preclinical and clinical studies demonstrated the beneficial use of DEX as a neuroprotective agent against cerebral ischemia, TBI, spinal cord injury, neurodegenerative diseases, and postoperative cognitive disorders [13].

**Figure 1:**
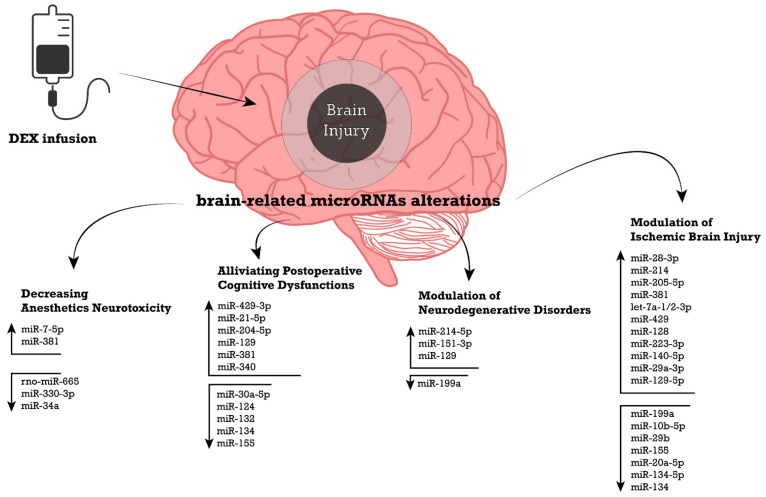
Changes in brain-related miRNAs expression after DEX treatment associated with neuroprotective effects in brain injuries. Upward and downward arrows represent the up-/down-regulation of miRNAs in response to DEX treatment. References: Decreasing the Anesthetics Neurotoxicity: [31,69,144,145]; Alleviating Postoperative Cognitive Dysfunctions: [28,70,74,75,76,146]; Modulation of Neurodegenerative Disorders: [29,71,147,148]; Modulation of Ischemic Brain Injury: [30,72,73,83,149,150,151,152,153,154,155,156,157,158,159,160,161,162]; Isolation of RNA from hippocampal neuron cells and brain homogenate was performed by using Trizol reagent method and total RNA was measured by using small RNA sequencing or real-time quantitative PCR (RTqPCR). The RNA quality and quantity of the RNA were analyzed with a spectrophotometer. The differentially expressed microRNAs were screened out by using Microarray analysis. In this context, the experimental studies aimed to investigate the neuroprotective potential of DEX by targeting miRNAs expression have been addressed to four topics, which will further explore: (1) modulation of ischemic brain injury, (2) decreasing the neurotoxicity of anesthetics, (3) reducing the postoperative cognitive dysfunction, including delirium or cognitive dysfunction, and (4) modulation of neurodegenerative diseases.

Furthermore, emerging preclinical studies have confirmed the hypothesis that DEX regulates miRNA expression in brain injuries (Figure 1). An in-depth understanding of the miRNA expression profile upon DEX administration is essential for emerging new circulatory biomarkers to estimate the neuroprotective potential of DEX, as well as developing new miRNAs-based therapeutics, which could enhance DEX’s pharmacological profile in protecting the brain.

### 6.2. Modulation of Ischemic Brain Injury

DEX activation could affect miRNA-mediated neuropathological responses under ischemic conditions by regulating direct or indirect pathways. There are several hypotheses suggesting that DEX treatment can affect miRNA-mediated neuroinflammatory and apoptotic responses, including the circ-CDR1as/miR-28-3p/TRAF3 pathway in mouse hippocampal neuronal cells exposed to H/R insult, miRNA-214/ROCK1/NF-κB pathway in rat cerebral ischemia-reperfusion injury (CI/RI), SNHG11/miR-324-3p/VEGFA and miRNA-10b-5p/BDNF pathway in MCAO ischemic rat model and neuron cell induced to OGD/R dysfunction.

As an endogenous RNA, which competes with miRNAs for the post-transcriptional regulation role, circ-CDR1as mediate cell viability and apoptosis, being involved in numerous nervous system conditions, including cerebral I/R, PD, and AD [144,145,146,147]. TRAF3 belongs to the TRAF adaptor protein family, regulating several cellular activities in multifold disorders and promoting apoptosis and inflammatory responses in vivo and vitro models of I/R [148]. Treatment with DEX after hypoxia-reperfusion injury induces neuronal protection by up-regulating miR-28-3p expression, and attenuating circ-CDR1as expression, thus reversing inflammatory responses and apoptotic rate in hippocampal neurons. Moreover, by upregulating miR-28-3p, DEX could mitigate TRAF3 overexpression, which has been associated with negative responses to brain I/R injury [148].

Liu et al., demonstrated the therapeutic effects of DEX in protecting neurons against ischemic damage by overexpressing miR-214, thus disrupting ROCK1 expression and dampening of NF-κB pathway, which are responsible for neuroinflammatory responses [149]. The level of miR-205-5p has been found to be low-expressed in a rat I/R injury model after MCAO and cultured hippocampal cells exposed to H/R injury [30]. Glutathione levels, altered malondialdehyde levels, and cytokine production have been shown to be attenuated upon DEX treatment [150]. DEX could alleviate neuroinflammation and oxidative stress associated with I/R injury by upregulating miR-205-5p, thereby decreasing HMGB1 expression and increasing anti-oxidative protein levels in rat brains [30].

Both DEX and miRNAs pose important roles in apoptotic signaling pathways by regulating many pro-apoptotic proteins. For instance, DEX negatively regulated cleaved caspase-3 and increased Bcl-2 expression in H_2_O_2_-induced dysfunction of PC12 cells [151]. DEX confers protection against hypoxia-induced injury in PC12 cells by inhibiting miR-134, thus reversing the increased level of cleaved caspase-3 and decreased Bcl-2 expression, related to elevated apoptotic rate [151].

Small nucleolar RNA host gene 11 (SNHG11) has been revealed to be significantly expressed in vivo and in vitro models of I/R injury, with its overexpressed level being associated with increased cell apoptosis and reduced cell viability [152]. I/R injury resulted in a low expression level of miR-324-3p, with upregulated SNHG11 and VEGFA levels in OGD/R induced neuronal injury on SH-SY5Y cells [152]. In this context, miR-324-3p targets SNHG11 and VEGFA to strengthen the neuroprotective effects of DEX in OGD/R-induced neuronal injury [152]. By inhibiting miR-199a expression, DEX exerts neuroprotection in rat CI/RI, restoring neuronal structure and improving modified neurological and cognitive functions.

By promoting neural stem cell proliferation and differentiation to neurons miR-381, its regenerative potential in multiple neurological disorders, such as AD, HD, PD, and spinal cord injury is revealed [153]. Interferon regulatory factors 4 and 5 (IRF5, IRF4) regulatory axis modulates microglial pro- and anti-inflammatory responses, corresponding to different cytokine production. IRF4 has been shown to regulate anti-inflammatory pathways related to microglia activation in the ischemic stroke model [154]. Treatment with DEX after MCAO rat models and primary neuron cells exposed to OGD increased miR-381 expression, which was associated with declined IRF4–IL-9 expression, leading to reduced apoptosis rate and improved neurological function [73].

BDNF, a crucial growth factor in the differentiation and survival of neurons during the development stage of the brain, prevented neuronal death in several hypoxic or excitotoxic models in the neonatal animal brain [155,156]. In a mouse model of brain excitotoxicity, DEX and BDNF exerted neuroprotective effects via the ERK1/2 pathway, DEX increasing the astrocyte expression of BDNF by subsequent activation of astrocyte’s α2-adrenergic receptors [155]. Upregulation of miR-10b-5p has been associated with neuronal apoptosis in ischemic brain injury by negatively regulating BDNF expression, with reversing the miR-10b-5p expression upon DEX treatment [157]. The protective mechanism induced by DEX has been mediated by the SNHG16-miR-10b-5p-BDNF pathway [157].

DEX poses important regulatory roles in hypoxic/ischemic injury by regulating the expression of inflammatory and pro-apoptotic factors in the neonatal brain [150,158].

In hypoxic ischemic brain damage (HIBD), MALAT-1 was highly expressed in the mice brain, leading to downregulation of miR-429 and ultimately increased WNT1 expression, which stimulates apoptosis of hippocampal neurons [159]. Otherwise, MALAT-1 silencing enhances the neuroprotective effect of DEX on HIBD by up-regulating miR-429, subsequently leading to the downregulation of WNT1 [159]. DEX elicited neuroprotective effects in neonatal ischemic brain injury by increasing expression of miR-128, co-treatment with both DEX and ago-miR-128 significantly increasing neuroprotection against brain injury and decreasing nerve cell apoptosis rate [83]. Moreover, upregulation of miR-128 promoted by DEX sustained down-regulating of WNT1, which will further lead to a decreased apoptotic rate of neurons [83].

In H_2_O_2_-induced neuronal injury, DEX promoted the expression of miR-223-3p and inhibited its target TIAL1. Pretreatment with DEX of neuroblastoma cells exposed to OGD has been proved to inhibit cell apoptosis, and sustain cell proliferation, possibly mediated by suppressing miR-29b [160]. In H_2_O_2_-induced injury, DEX protected PC12 cells from oxidative damage by down-regulating miR-199a, thereby promoting an increase in HIF-1α and activating the neuroprotective pathways of PI3K/AKT/mTOR and Wnt/b-catenin pathways [161].

ERK pathway serves important regulatory roles in neuron proliferation, and apoptosis, participating in some of the signaling cascades of DEX to induce neuroprotection [162,163]. According to Zhu et al., downregulation of miR-155, mediated by DEX treatment in OGD/R injury of rat hippocampal neurons, promotes the ERK1/2 pathway and enhances matrix metalloproteinases, thereby inhibiting apoptosis and oxidative stress [164].

Histone Deacetylase 4 (HDAC4) mediates epigenetic mechanisms and acts as transcriptional repressor to maintain normal transcriptional gene transcription. In the brain, HDAC displays neuroprotective effects in AD, and ischemic stroke, and interacting with miRNAs [165]. MiR-29a-3p promotes the neuroprotective effects of DEX on HIBD rats by interfering with neuron inflammation and apoptosis in the hippocampal region, by inhibiting HDAC4 [72].

Maternally expressed gene 3 (MEG3), long noncoding RNA, emerged as a competing endogenous RNA for miRNAs in experimental disease models. MEG3 suppressing, or miR-129-5p upregulation enhanced the neuroprotection effect of DEX on neonatal mice with HIBD, hence reducing neuronal apoptosis rate and sustaining cerebral function recovery [166]. Microglial activation represents the first initiator of neuroinflammation in response to ischemic brain injury, subsequently leading to neuronal damage [167,168]. Moreover, microglial activation might be modulated by miRNAs [24]. In spinal cord ischemia-reperfusion injury, DEX suppressed the activation of microglial cells via upregulation of let-7a-1/2-3p and downregulation of the HMGB1 pathway, model, which has been related to improved nerve motor function [169].

### 6.3. Decreasing the Neurotoxicity of Anesthetics

In neurotoxicity models of anesthetics, DEX could modulate pro-apoptotic and anti-apoptotic signaling pathways [69]. DEX treatment regulates neuron apoptosis against anesthetics-induced neurotoxicity in multiple toxicity models of sevoflurane, ketamine, and isoflurane, in both, immature and adult brains [170,171,172]. Besides the anesthetic toxicity associated with surgical procedures, oxygen, used mainly in neonatal intensive care, has been associated with disruption of intracellular hemostasis reaction and subsequently activation of oxidative stress and inflammation, which were responsible for long-term cognitive dysfunction and neurodegeneration [150]. In a hyperoxia induced-neurodegeneration model, DEX promotes neuroprotective effects on neonatal rat brain injury by restoring altered glutathione levels and suppressing lipid peroxidation, counteracting deleterious effects of oxidative stress [150].

Furthermore, an alteration in brain-related miRNAs has been shown after anesthetic exposure [173]. Ropivacaine, used as a local anesthetic, could exert neurotoxic effects on neurocyte cells [174]. In a model of ropivacaine-induced neurocyte injury, ropivacaine attenuates miR-381 expression and enhances LRRC expression, inducing apoptotic and anti-proliferative effects on neurocyte cells [31]. Cotreatment with DEX upregulated miR-381, with suppression of LRRC and activation of SDF-1/CXCR4 pathway [31]. In human neuroblastoma SH-SY5Y cells, DEX reversed the apoptotic effect of bupivacaine in a dose-dependent manner by decreasing the levels of pro-apoptosis associated proteins Bax and cleaved caspase-3 and reducing ROS production [69].

Parthanatos, a caspase-independent cell death was linked to the overactivation of PARP-1 [175]. The involvement of parthanatos in brain disorders has been revealed in cerebral ischemia, glioma cells, and other CNS-associated pathological processes [176,177]. To pose the DEX mediated effect against nervous cells parthanatos, a toxicity model of Bupivacaine on human neuroblastoma cells was created [69].

Thus, DEX inhibited bupivacaine-induced PARP1 expression and increased miR-7-5p expression, attenuating the oxidative stress. Moreover, miR-7-5p upregulation protects against parthanatos by stabilizing the mitochondrial membrane potential and antioxidative enzymes, which were disrupted by bupivacaine [69]. Some of DEX’s neuroprotective properties in the neurotoxicity model of sevoflurane- were mediated by miR-330-3p/ULK1 pathway, thus inhibiting cell apoptosis and facilitating mitophagy [178]. MiR-129 has been shown to target Toll-like receptor 4 (TLR4). Up-regulation of miR-129 in SEV-induced post-operative cognitive dysfunction (POCD) rats inhibited TLR4 expression and prevented NF-κB p65 phosphorylation, thus alleviating cognitive impairments in rats, and hindering neuronal apoptosis [70].

### 6.4. DEX-Mediated Neuroprotection against Postoperative Cognitive Dysfunctions

Several neurodegenerative diseases are linked to neuroinflammation, with a strong correlation between neuroinflammation and increased peripheral proinflammatory cytokines being evidenced [179,180]. During surgery, the release into circulation of a large amount of pro-inflammatory cytokines, e.g., IL1-β and TNF-α, and subsequent activation of the immune system were correlated with neuronal dysfunction and death, leading to postoperative cognitive impairments [76].

Neuroinflammation induced by LPS treatment is followed by changes in the expression profiles of miR 124, 132, 134, and 155 in a time-dependent manner in different tissues, including the hippocampus, cortex, and plasma [76]. Treatment with DEX attenuates LPS-induced upregulation expression of these miRNAs at different time points, with different and fluctuated levels of miRNAs expression [76].

DEX administrated alone could significantly change the expression levels of miRNAs in the brain tissue under normoxic conditions, with more enhanced regulation of miRNAs in the neuroinflammatory context [76]. Therefore, DEX alone promotes the same ‘increasing’ trend in the expression of miR-124, 132, 134, and 155, as in the LPS-induced neuroinflammation model [76]. MiR-155 has been revealed to negatively regulate the BBB in a rat model of LPS-induced neuroinflammation [181]. Otherwise, DEX attenuated disruption of BBB during neuroinflammation [182]. In this context, Paeschke et al., hypothesized a possible involvement of DEX in attenuating BBB disruption by modulating miR-155 [76]. In the blood samples of 40 patients who underwent off-pump coronary artery bypass grafting, circulating miRNAs exhibited downregulated levels of miR-320 after adjunct anesthesia with DEX, with high expression of neuroglobin (NGB), suggesting the role of miR-320/NGB pathways in enhancing the proliferation activity of neuronal cells, associated with reduced POCD [77].

Recent findings provide insight into the neuroprotective and organ-protective mechanism of DEX by restoring the autophagic flux in the spleen, attenuating the PARP cleavage induced by LPS in the spleen, and regulating the cholinergic anti-inflammatory pathway [75]. DEX induces neuroprotection by attenuating LPS-induced upregulation of miR-30a-5p and downregulation of miR-21-5p and miR-204-5p in the hippocampus. Interestingly, DEX did not suppress the alteration of miRNA expression in the cortex induced by LPS, but regulated the expression of miR-204-5p, miR-Let-7a-5p, and miR-21-5p in the spleen [75].

DEX treatment downregulates miR-29b expression, thus affecting cell viability, and inhibiting cell apoptosis in the OGD-induced neuronal injury [160]. Up-regulation of miR-340 expression in primary microglia cultured cells (BV2 microglia cells) under treatment of LPS enhanced the anti-inflammatory effect of DEX via inhibiting the NF-κB pathway. Furthermore, DEX promotes phagocytosis in LPS-induced microglia cell dysfunction [28]. In POCD-operated mice anesthetized with sevoflurane, DEX administration resulted in enhanced miR-381 expression and inhibition of EGR1/p53 pathways, related to decreased rate of hippocampal neuron apoptosis, DNA damage, neuroinflammation, and cognitive impairment [74].

### 6.5. Modulation of Neurodegenerative Disorders

Neuroinflammation triggered by abnormal protein complexes’ formation represents a critical step in the initiation and progression of neurodegenerative disorders [183]. The neuroprotective therapy aimed to counteract the immunological and neurodegenerative reactions in AD, PD, MS is based on the modulation of microglial cell activation and regulation of the releasing of pro-inflammatory mediators [184].

The anti-inflammatory and immunomodulatory effects of DEX in neurodegenerative disease models proceed by suppressing both cytokine production and microglia activation [185]. MOG-induced animal experimental autoimmune encephalomyelitis (EAE) reproduces infiltration of spinal cord with microglia cells, clinically mimicking MS. In C57BL/6 mouse-induced EAE, DEX reduces microglial infiltration in the spinal cord, by suppressing the expression of CXCR7, a chemokine receptor expressed on the surface of microglia, thus inhibiting chemotaxis of microglia triggered by SDF-1 or I-TAC. By binding to α2 AR, DEX induces desensitization of microglial CXCR7 in the mouse model of EAE. Thus, DEX attenuated the clinical severity of EAE, suggesting that MS could benefit from the immunopharmacological effects of DEX [185].

Inflammasomes, multiprotein complexes responsible for the synthesis of pro-inflammatory cytokines, IL-1β and IL-18, in response to ROS production, modulate the initial stages of neuroinflammation during multiple neurodegenerative diseases (AD, TBI, MS) [186,187]. Nod-like receptor (NLR) family proteins, essential components of the inflammasome, are specific sensors or receptors which recognize multiple damage signals, such as pro-caspase-1, and ASC (the apoptotic speck-like protein containing a caspase recruitment domain) [188,189]. In neuroinflammation models, NLRP inflammasome induces activation of caspase-1, thus releasing IL-1β and IL-18, which are responsible for further recruitment of microglia and astrocyte cells [186,190]. In vivo rat model of rheumatoid arthritis (RA), DEX suppresses NLRC5, therefore reducing cytokine levels of IL-1β, IL-6, IL-17A, and TNF-α. In human RA, DEX inhibits migration, invasion, and inflammation of fibroblast-like synoviocytes by decreasing the NLRC5 level, which will further inhibit NF-κB pathway [191]. Therefore, modulation of inflammasome activation by DEX treatment could be a promising neuroprotective strategy in neurodegenerative diseases by inhibiting NLRP inflammasomes.

Macrophage migration inhibitory factor (MIF), an immunoregulatory cytokine, produced by both innate and adaptive immune systems has been shown to be involved in the pathogenesis of multiple autoimmune disorders, exerting both immunoinflammatory and immunomodulatory effects in neurodegenerative disease models [192,193]. Immunopharmacological effects of MIF include increasing the TLR4 expression, inducing phagocytosis, and Th1 immune response, and stimulating cytokine-producing [194,195]. MIF poses enzymatic, endocrine, and cytokine-like roles, by binding to CD74 cell surface receptor and C-X-C chemokine receptors (CXCR2, CXCR4, CXCR7) [195]. MIF primarily displays pro-inflammatory effects, by activating pro-inflammatory mediators, such as IL-6 and TNF-α, and also inducing activation of inflammasome [196]. In a mouse model of PD, the upregulated profile of MIF exerts an antiapoptotic and autophagy-inducer role, suggesting the beneficial role of MIF in PD [197]. D-dopachrome tautomerase (D-DT) (also referred to as MIF2), the structural homologue of MIF, exerts synergic functions similar to MIF, both contributing to the progression of MS in males [198]. Benedek et al., found high levels of MIF and D-DT in MS male and female patients, with higher levels of CD74 in females than in males with increased MS disease severity [198]. In an EAE mouse model, mice lacking MIF or D-DT have been shown less severe symptoms of EAE. As MIF and D-DT are emerging as disease modifiers in MS, immunopharmacological therapy aimed to target CD74, the transducer of MIF signaling, might be a promising neuroprotective therapy in inhibiting the clinical progression of MS [198,199]. Given the anti-inflammatory and immunomodulatory profile of DEX, future studies to evaluate the impact of DEX on MIF and D-DT will be needed.

Moreover, immunoinflammatory disorders such as major depressive disorder characterized by high levels of inflammatory biomarkers have been shown reduced MIF levels after antidepressants, suggesting the pathogenic role of MIF in the induction and maintenance of these disorders [200].

Although multiple studies have shown the effect of DEX on decreasing pro-inflammatory cytokines (IL1-β and TNF-α) in animal models of neuroinflammation and neurodegenerative disorders, there is no data regarding the influence of DEX on anti-inflammatory cytokines that might play a protective role in these diseases [71,76]. Larger studies to explore the immunomodulatory role of DEX on anti-inflammatory cytokines associated with neuroprotection during neurodegenerative disorders including TGF beta, IL1 receptor antagonist, IL10, IL13, IL35, and IL37 should also be proposed.

Increasing evidence supported the contribution of miRNA dysregulation in the development and progression of neurodegenerative diseases such as AD, ALS, and HD [201,202,203].

In post-mortem analysis of PD patients, Hoss et al., have found a number of 125 miRNAs to be significantly altered in the prefrontal cortex [25].

In an APPswe/PS1dE9 mouse model of AD, DEX administration decreases the apoptosis rate of hippocampal neurons, and neuroinflammatory and oxidative stress-related processes, which were associated with up-regulated miR-214–5p and down-regulated SUZ12 [71]. The expression level of Yes-associated protein 1 (YAP1) and Jagged 1 (JAG1), have been shown to be up-regulated in AD patients, with miRNAs modulating the interaction between YAP1 and JAG1 [204,205,206]. In Aβ1-42-injected mice, DEX improved hippocampal neuron apoptosis and attenuated cognitive impairment by enhancing miR-129 which subsequently disrupt the interaction between YAP1 and JAG1, protecting against AD [207]. Upregulating miR-151-3p promotes anti-apoptotic and anti-oxidative effects of DEX on Aβ-treated neuronal cells by modulating DAPK-1 and TP53 signaling pathways [29]. Interestingly, DEX stimulates pro-apoptotic signal by activation of the ERK1/2 pathway, but TP53, the downstream effector of this pathways, abrogated this effect, suggesting a balance between pro-apoptotic and anti-apoptotic pathways [29].

**Figure 2:**
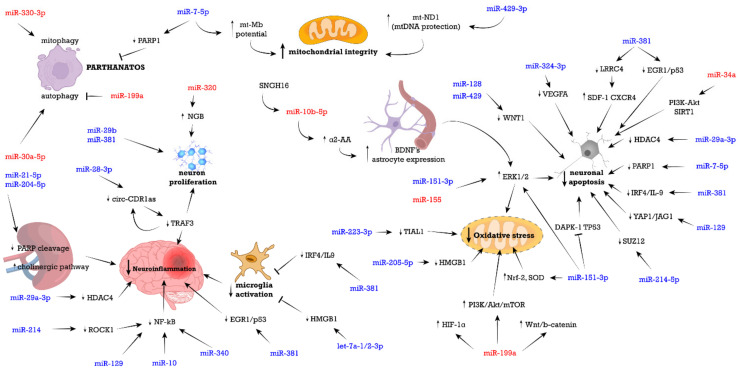
Molecular pathways shared between miRNAs and their associated signaling pathways for promoting dexmedetomidine’s neuroprotective effects. Brain injuries that involved miRNA regulation, in which dexmedetomidine poses neuroprotection refer to: ischemic brain injury, anesthetics-induced neurotoxicity, postoperative cognitive dysfunction, and neurodegenerative diseases. Dexmedetomidine regulates a variety of neuropathogenic pathways which involves miRNAs, including: neuroinflammation, oxidative stress, and apoptosis, with a few contributions of mitophagy, autophagy, parthanatos, and mitochondrial-associated processes. miRNAs color-coded in blue represent those that are upregulated after DEX treatment in brain disorders, and miRNAs in red represent those that are downregulated after DEX treatment.

An overview of the miRNAs involved in the neuroprotective mechanisms of DEX is illustrated in Figure 2.

## 7. MiRNAs in Pharmacological Response of Dexmedetomidine and Drug Response Variability

### 7.1. Involvement of MiRNAs in Drug Response Variability

miRNAs are increasingly being researched in the pathogenesis of multiple diseases, but the study of their involvement in drug response variability is essential to strengthening the effectiveness of the existing therapies [208].

Interindividual variability of drug response influences the efficacy and toxicity of drugs, and is controlled by both genetic and environmental factors [209,210]. Pharmacoepigenomics identifies the variability in gene expression related to drug response, and proceeds via methylation, histone modification, and miRNAs at the transcriptional and post-transcriptional levels [211].

Pharmacological responses of drugs are also controlled by miRNAs, as different expression levels of miRNAs have been revealed after several drugs’ administration [212].

MiRNAs could affect epigenetic regulation of genes in the expression of drug transporters, such as ABC and SLC transporters, and drug-metabolizing enzymes (DMEs), including cytochrome P450s (CYP450s) [212]. CYP2C9 responsible for the metabolism of warfarin and phenytoin, has been revealed to be suppressed after overexpression of miR-128-3p in HepaRG cells [213]. MiR-297 sensitizes colorectal multidrug resistance cancer cells to vincristine, oxaliplatin, and 5-fluorouracil, by reducing ABCC2 protein expression [214]. Another mechanism that could explain the regulation of drug transporters and DMEs consists in modulation of the expression of nuclear receptors, such as xenobiotic-sensing nuclear receptors family such as pregnane X receptor (PXR), hepatocyte nuclear factor 4 alpha (HNF4α), peroxisome proliferator-activated receptor alpha (PPARα) and nuclear hormone receptors, including glucocorticoid receptor (GR), estrogen receptor 1 (ESR1) [212]. The miRNAs expression profiling has shown significant changes after drug treatment. For instance, increases in serum levels of miR-451a and decreases in the serum levels of miRNA-34a-5p and miRNA-221-3p have been revealed in 84 depressed patients after treatment with paroxetine [215].

Besides this, miRNA polymorphisms or miRSNPs play critical roles in gene regulation, consisting of polymorphisms at the binding sites of miRNAs or at the level of miRNA sequences during biogenesis processes [212].

Numerous polymorphisms of miRNAs emerged as potential prognostic biomarkers and disease-modifying agents in cancer chemotherapy. LCS6 rs61764370 T > G, the polymorphism within let-7 binding site in the KRAS gene was associated with poor prognosis and response to cetuximab-irinotecan therapy in metastatic colorectal cancer [216]. Two polymorphisms, pri-miR26a-1rs7372209, and pri-miR-100rs1834306 were associated with different responses to chemotherapy treatment in metastatic colon cancer in response to 5-fluorouracil and irinotecan [217].

MiRNAs might be suitable biomarkers of individual drug response due to their stability in many body fluids, as well as their simple availability and reliable measurement [208]. Numerous promising genomic biomarkers to estimate drug responses are emerging for several disorders. For instance, miR-301 levels were elevated in non-respondent schizophrenic patients after haloperidol treatment [218]. In rheumatoid arthritis (RA) patients, analysis of blood samples indicated a high level of miR-125b-5p expression in good response patients, outlining the role of miR-125b-5p as predicting biomarker in response to Rituximab treatment [219].

### 7.2. MiRNAs in Dexmedetomidine’s Pharmacological Profiles

DEX exhibited significant differences in miRNAs expression, emerging promising miRNAs biomarkers to identify the interindividual variability of drug response. Mounting evidence supports the role of DEX in modulation of miRNAs expression for promoting specific effects in multiple organs, but the role of miRNAs in the variability of drug response was not fully explored.

A higher miRNA-183 expression in peripheral blood of 80 patients who underwent laparoscopic cholecystectomy and oophorocystectomy has been confirmed after treatment with DEX in day surgery, which was correlated with improving the remifentanil-related hyperalgesia [220]. In AD mice, miRNA-214-5p enhances the cerebral protection of DEX against neuronal injury, attenuating the cognitive impairment of AD mice [71]. Moreover, miRNA-151-3p promoted anti-apoptotic and anti-oxidative mechanisms against Aβ-treated neuronal cells, by modulating DAPK-1 and TP53 signaling pathways [29].

α2-adrenergic receptors (α2AAR) play significant regulatory roles in depressive responses, including postpartum depressive symptoms (PDS) pathophysiology, which were associated with an elevation in presynaptic α2AAR.

miRSNPs poses also an important role in α2AAR gene transcription, with important clinical significance in decreasing PDS incidence [221]. In a randomized clinical study, DEX attenuates PDS incidence in 600 women following cesarean section, by stimulating α2 adrenergic receptors [222].

α2AAR gene polymorphisms have been shown to play important roles in 103 PDS patients, who underwent cesarean section. α2AAR rs13306146 A > G polymorphism resulted in a decreased binding ability of miR-646 to the α2AAR gene at the 3′UTR, leading to a reduced α2AAR gene expression [221]. Cai et al., in a predictive study of response to DEX treatment, proposed miR-30a-5p, miR-101-3p, miR-140-3p, and miR-141-3p as predictive serum biomarkers for preoperative sedation outcome in 133 pediatric patients [33]. A higher plasma level of miR-101-3p and miR-140-3p was observed in respondents, while a downregulated expression of miR-101-3p and miR-30a-5p was associated with DEX adverse effects, i.e., hypotension and bradycardia [33]. Another study identified 12 differentially expressed human miRNAs in three patients after DEX treatment, five of that were upregulated (hsa-miR-4508, hsa-miR-novel-chr8_87373, hsa-miR-30a3p, hsa-miR-novel-chr16_26099, hsa-miR-4306 and seven downregulated (hsa-miR-744-5p, hsamiR-320a, hsa-miR-novel-chr9_90035, hsa-miR-101-3p, hsa-miR-150-5p, hsa-miR-342-3p, and hsa-miR-140-3p) [223]. In terms of DMEs of DEX, miR-141-3p down-regulates UDP-Glucuronosyltransferase (UGT) 1A4 enzyme UGT1A4, an important metabolizer of DEX treatment. A summary of promising miRNAs described so far in clinical setup, which might be used as predictive human biomarkers for DEX response can be viewed in Table 3.

Given the limitations of genomic analysis studies, aimed to estimate the circulating level of miRNA in response to DEX treatment, such as software predictions, lack of healthy sample subjects, and a more in-depth analysis of differential expression of miRNA depending on conditions of the health of patients will need to be conducted.

Therefore, miRNAs might be promising tools in estimating the interindividual variability of DEX response and also the safety and efficacy of DEX treatment in both healthy and disease conditions. Analyzing the circulating miRNAs in clinical studies in different conditions of the health, could translate precise genomic biomarkers of DEX response into clinical setups.

## 8. Potential Clinical Applications and Therapeutic Targets

With the knowledge advancement in the field of miRNAs, more and more studies outline the potential of miRNAs as peripheral biomarkers and disease-modifying agents in multiple brain disorders [137,224].

The applicability of miRNAs-based biomarkers in brain disorders has been ascertained in plasma, serum, cerebrospinal fluid, and other biological fluids [225]. Due to their stability in serum and other body fluids, and their correlation with disease prognosis, miRNAs were proposed as predictive biomarkers in disorders and drug response [208].

Understanding the role of miRNAs involved in the variability of drug response in terms of DEX efficacy and safety might strengthen the existing pharmacological profile of DEX. Moreover, targeting the miRNAs involved in the protective effects of DEX in multiple disorders, using specific miRNAs-based agents, termed agomirs and antagomirs, represents a promising therapeutic strategy for multiple clinical applications of DEX. Thus, we could supplementarily regulate the existing miRNAs regulated by DEX itself, therefore achieving a powerful effect of DEX against brain injuries.

On the other hand, we could modulate miRNAs responsible for DEX pharmacological responses, by using agomirs and antagomirs, reducing adverse effects and strengthen the existing DEX effects, thus achieving a personalized therapy for patients, which are candidates for DEX therapy. Thus, downregulating the overexpressed miRNAs associated with non-respondent patients after DEX therapy, using miRNAs blockers (antagomirs), or miRNA mimics (agomirs) might be a promising therapeutic tool.

The translation into practice of miRNAs-based agents in clinics for DEX treatment comes with pearls and pitfalls. From the miRNAs expression changes in a time-related manner to the variations depending on the intensity of the stimulus to the age and sex differences, all of which are part of the hard-to-control variables of the expression profiling of miRNAs [226]. Another impediment stems from the necessity of achieving an effective delivery system to address improper distribution, accumulation in non-target organs, non-specific absorption, toxicity, and rapid circulation degradation by systemic [227,228,229].

Besides the aforementioned considerations, we must take into consideration the limitations of animal studies, including low-quality scores, lack of data from females, old animals, and experimental models of comorbid animals [230].

More research studies will be needed to elucidate the molecular mechanisms of DEX in the neuroprotection context, and emerging new signaling pathways which could interact with miRNAs. Moreover, advancing in miRNA technology for developing new stable and feasible miRNAs-based biomarkers and therapeutic agents will further reinforce the existing DEX therapy against brain injury and avoid variability of DEX response and adverse effects. A better understanding of miRNAs-based gene regulation of DEX treatment could strengthen the existing neuroprotective effects of DEX treatment against brain injuries and could also provide future directions in individualized drug therapy.

## Figures and Tables

**Table 1:** Main effects of dexmedetomidine administration.

Effect	Mechanism/Consequences	References
Sedative	activates central pre- and postsynaptic α2ARs in the locus coeruleus decreases norepinephrine release mimics physiological sleep	[39]
Analgesic	activates α2ARs in the spinal cord–dorsal horn (locus coeruleus) decreases the release of substance P	[54,55]
Anti-inflammatory	↓ TNF-α, IL-6, IL-8, IL-1β ↑ IL-10 ↑ NF-κB and CD4 cells ↓ CD8 cells ↓ plasma level of epinephrine, norepinephrine, and cortisol increases the degradation of NLRP3 inflammasome (a molecule with a role in activating caspase-1 and secreting proinflammatory cytokines such as IL-1β or IL-18)	[49,56,57,58,59]
Antioxidant	decreased ROS and increased SOD, GSH, and CAT after LPS or acetaminophen-induced oxidative stress	[60,61]
Increases blood pressure after rapid bolus injection	changes the expression α_2_ AR from the subtype α2A to a1 and α2B. α2A ARs are involved in vasodilation by NO production activates phospholipase A and increases the vascular response to angiotensin and catecholamines.	[54,62]
Decreases blood pressure after intravenous continuous infusion	activates presynaptic α2ARs decreases norepinephrine release decreases sympathetic outflow	[63,64]

Abbreviations: α2ARs, α2 adrenergic receptor; CAT, Catalase; CD 4, 8, cluster of differentiation 4, 8; GSH, Glutathione; IL-6, -8, -1β, 10, 18, Interleukin 6, -8, -1β, 10; LPS, Lipopolysaccharide; NF-κB, nuclear factor kappa-light-chain-enhancer of activated B cells; NLRP3, NLR family pyrin domain containing 3; NO, nitric oxide; ROS, Reactive oxygen species; SOD, Superoxide dismutase; TNF-α, Tumor necrosis factor-α.

**Table 2:** End-organ effects of DEX.

Study	miRNA	Role of miRNA	Effect of DEX on miRNA	Biological Effect of miRNA	References
In vitro Human neuroblastoma SH-SY5Y cells—bupivacaine-induced Neuron injury	miR-7-5p	Improved mitochondrial membrane potential Increased antioxidative enzymes and reduced apoptosis Impaired bupivacaine-induced parthanatos	Upregulated	Inhibiting PARP1 ↓ SOD1, CAT ↑ GPx ↓ Bax, cleaved caspase 3	[69]
In vivo Sprague-Dawley rats—postoperative cognitive dysfunction induced by sevoflurane	miR-129	involved in cognitive dysfunction-related diseases	Increases expression in hippocampus and cortical neurons	Inhibiting TLR4 and NF-κB p65 phosphorylation	[70]
In vivo: APPswe/PS1dE9-induced AD mice	miR-214-5p	involved in reducing apoptosis neuroinflammation and oxidative stress of hippocampal neurons Attenuating cognitive impairments of AD mice	Upregulated expression in hippocampal neurons	Downregulated SUZ12	[71]
In vivo Newborn Sprague-Dawley rats—induced HIBD	miR-29a-3p	Elevated MiR-29a-3p reduces inflammation and apoptosis rate of hippocampal neurons increase spatial learning and memory abilities Improved brain atrophy and alleviated pathological changes in ischemic rat brain	Upregulated	Negatively regulating HDAC4 ↓ IL-6, IL-8, and TNFα ↑ IL-10	[72]
In vivo Wistar rats—induced CI/R injury In vitro Rat hippocampal neurons cells	miR-205-5p	May inhibit the inflammatory response and oxidative stress reduced brain infraction volume, improved neurological function score in acute cerebral ischemia induced in rats	Upregulated expression in hippocampal neurons	Negatively regulating HMGB1 ↑ SOD, ↓ ROS, MDA ↑ Nrf2, GR, GPX, HO-1, CAT ↓ IL-1β, IL-6, and TNF-α	[30]
In vivo Sprague-Dawley rats —CI/R injury In vitro Primary hippocampal neuron cell culture exposed to OGD/R treatment	miR-381	inhibited inflammation response and neuron cell apoptosis improved neurological function	Upregulated	Inhibiting IRF4-IL-9	[73]
In vivo C57BL/6 mice—sevoflurane-induced POCD In vitro primary hippocampal neurons from newborn mice		involved in apoptosis, neuroinflammation, and DNA damage repair Alleviates Cognitive Dysfunction	Upregulated	Inhibiting EGR1/p53 Disrupts interaction of EGR1 with p53 Decreasing EGR1-mediated p53 transcription	[74]
In vitro cell culture (Pheochromocytoma cell line—PC12)—ropivacaine-induced neuronal injury		may reduce the proliferation and apoptosis of ropivacaine-induced PC12 cells	Upregulated	Negatively regulating LRRC4 expression ↓ Cleaved-Caspase-3 ↑ Bcl-2	[31]
In vivo Wistar rats—lipopolysaccharide (LPS)-induced inflammation	miR-21-5p	involved in inflammation, autophagy, and apoptosis	Decreased the expression in the spleen and hippocampus	Attenuated PARP cleavage in the spleen	[75]
	miR-204-5p				
	miR-30a-5p				
In vivo Wistar rats—LPS-induced neuroinflammation	miR-124	involved in neuronal differentiation	Decreased the expression in the hippocampus and cortex	Targeted SHIP1, SOCS1	[76]
	miR-132	involved in the inflammatory response in alveolar macrophages potentiated cholinergic anti-inflammatory pathway			
	miR-155	involved in pro- and anti-inflammatory mechanisms			
In vivo 40 patients undergoing off-pump coronary artery bypass grafting	miR-320a	associated with cell proliferation	Decreased miRNA-320 expression level in the blood of patients after treatment with DEX	Stimulated NGB expression	[77]
In vivo Adult male Sprague-Dawley rats- chronic inflammatory visceral pain	miR-211	Down-regulated in TNBS-induced chronic inflammatory visceral pain	Upregulated after DEX treatment	Negatively regulating ERK expression ↓ IL-1β, TNF-α, and IL-6	[78]
In vivo Adult male Sprague-Dawley rats In vitro primary myoblasts from young mice In vivo Coopworth ewes sheep	rno-miR-434-3p	miR-434-3p protects myocytes from apoptosis	Upregulated	Targeted EIF5A G2E3, DCAF6, and TMEM68 ↓ caspases-3, −8 and −9	[79,80]
	rno-miR-3596d			Targeted G2E3, DCAF6, TMEM68, ATAD2B, NPY1R, SRSF1, ITGA6, MORC3, and RSF1	
	rno-miR-496-5p			Controlled mTOR pathway Targeted ATAD2B, NPY1R, SRSF1, ITGA6, MORC3, and RSF1	
	rno-miR-7a-2-3p			Negatively regulating PARP expression	
	rno-miR-702-3p				
	rno-miR-208b-3p	Upregulated in heart failure	Downregulated	Targeted CSNK2A2/NLK Promoted Wnt/β-catenin pathway ↑ Bcl-2	[79,81]
In vivo Sprague-Dawley rats—MI/R injury	miR-346-3p	miRNA-346 suppresses infarct size inhibits myocardial cell apoptosis may protect against MI/R injury	Upregulated the expression	Negatively regulated CAMK2D expression ↓ NF-κB ↓ NLRP3 inflammasome	[67]
In vivo LPS-induced acute lung injury of mice	miR-223-3p	The absence is associated with severe lung inflammation pulmonary up-regulation of this miRNA in mice may provide protection during acute lung injury due to various causes (eg. mechanical ventilation)	Upregulated	Negatively regulated HDAC4 expression ↓ TLR4 ↓ NF-κB	[82]
In vivo neonatal mice model—induced HIBD	mmu-miR-128	Increased the neuroprotective effects of DEX against ischemic brain injury attenuated nerve cell apoptosis enhanced learning and memory abilities reduced left-brain water content	Upregulated	Negatively regulating WNT1 expression	[83]

Abbreviations: AD, Alzheimer’s Disease; ATAD2B, ATPase Family AAA Domain Containing 2B; Bax, BCL2 Associated X, Apoptosis Regulator; Bcl-2, B-cell lymphoma; CAT, Catalase; CAMK2D, Calcium/calmodulin dependent protein kinase II delta; CI/R, cerebral ischemia/reperfusion; CSNK2A2, Casein Kinase 2 Alpha 2; DCAF6, DDB1 And CUL4 Associated Factor 6; EGR1, Early Growth Response 1; EIF5A, Eukaryotic Translation Initiation Factor 5A; ERK, extracellular signal-regulated kinases; G2E3, G2/M-Phase Specific E3 Ubiquitin Protein Ligase; GR, Glutathione reductase; GPX1, glutathione peroxidase-1; HDAC4, Histone deacetylase 4; HIBD, Hypoxic Ischemic Brain Damage; HMBG1, High mobility group box 1; HO-1, Heme oxygenase-1; IRF4, Interferon Regulatory Factor 4; IL-1β, -6, Interleukin -1β, -6; ITGA6, Integrin Subunit Alpha 6; LPS, Lipopolysaccharide; LRRC4, Leucine Rich Repeat Containing 4; MCAO, Middle Cerebral Artery Occlusion; MI/R, myocardial ischemia/reperfusion; MORC3, MORC Family CW-Type Zinc Finger 3; NGB, Neuroglobin; NLK, nemo-like kinase; NLRP3, NLR family pyrin domain containing; NPY1R, Neuropeptide Y Receptor Y1; NF-κB, nuclear factor kappa-light-chain-enhancer of activated B cells; Nrf2, Nuclear factor-erythroid factor 2-related factor 2; OGD/R, oxygen-glucose deprivation/Reperfusion; p53, Tumor protein P53; PARP1, Poly(ADP-ribose) polymerase 1; POCD, Postoperative Cognitive Disorder; ROS, Reactive oxygen species; RSF1, Remodeling And Spacing Factor 1; SHIP1, phosphatidylinositol-3,4,5-trisphosphate 5-phosphatase 1; SOCS1, suppressor of cytokine signaling 1; SOD, Superoxide dismutase; SRSF1, Serine And Arginine Rich Splicing Factor 1; SUZ12, Suppressor of ZESTE 12; TMEM68, Transmembrane Protein 68; TLR4, Toll-like receptor 4; TNBS, trinitrobenzene sulfonic acid, TNF-α, Tumor necrosis factor-α.

**Table 3:** Circulating human miRNAs expression profiling in DEX treatment, emerging as promising genomic biomarkers of DEX response in clinical studies.

miRNAs	Type of Study	Intervention	Observations	References
miR-320	40 patients	Off-pump coronary artery bypass grafting	Downregulated levels after treatment	[77]
miR-183	80 patients	Laparoscopic cholecystectomy and oophorocystectomy surgery	Overexpressed after treatment	[220]
miR-646--α2AAR s13306146 polymorphism	568 cesarean section patients	Chinese women who received cesarean section	miR-646 level in α2AAR s13306146 polymorphism affects α2AAR Postpartum Depressive Symptoms	[221]
miR-30a-5p, -101-3p, -140-3p and -141-3p	133 pediatric patients	Preoperative sedation for different procedures	Increased levels of miR-101-3p and 140-3p in respondents and downregulated levels of miR-101-3p and miR-30a-5p in hypotension and bradycardia patients	[33]
hsa-miR-4508, -novel-chr8_87373, -30a-3p, -novel-chr16_26099, -4306, -744-5p, -320a, -novel-chr9_90035, -101-3p, -150-5p, -342-3p, and-140-3p	three patients	Elective surgery	Five miRNAs upregulated (hsa-miR-4508, -novel-chr8_87373, -30a-3p, -novel-chr16_26099, -4306,) and seven miRNAs downregulated (hsa-miR-744-5p, -320a, -novel-chr9_90035, -101-3p, -150-5p, -342-3p, and -140-3p) after DEX treatment	[223]

Abbreviations: α2AAR, α2-adrenergic receptors; miRNAs, miRs; DEX, dexmedetomidine.

## Data Availability

Not applicable.
